# Research hotspots and trends of GLP-1 receptor agonists in the treatment of Parkinson’s disease: a bibliometric analysis

**DOI:** 10.3389/fnins.2026.1894856

**Published:** 2026-07-29

**Authors:** Ting Yu, Qingxin Mai, Qian Wang, Mingxia Pan, Yuanfang Xiong, Lingyu Kuang, Hanjiao Liu

**Affiliations:** 1 Shenzhen Hospital of Integrated Traditional Chinese and Western Medicine, Shenzhen, Guangdong, China; 2 School of Nursing, Fujian University of Traditional Chinese Medicine, Fuzhou, Fujian, China; 3 Shenzhen Clinical College of Integrated Traditional Chinese and Western Medicine, Affiliated to Guangzhou University of Chinese Medicine, Shenzhen, Guangdong, China

**Keywords:** Parkinson’s disease, GLP-1 receptor agonists, type 2 diabetes mellitus, GLP-1, bibliometric analysis

## Abstract

**Background:**

PD is still managed symptomatically with levodopa, and no disease-modifying therapy is available, making effective treatment difficult. In recent years, preclinical and some clinical evidence have shown that glucagon-like peptide-1 receptor agonists (GLP-1 receptor agonists, GLP-1 RAs) can cross to varying degrees the blood–brain barrier and exert neuroprotective effects, including anti-inflammatory, antioxidant, and insulin-sensitizing actions. They also reduce pathological protein aggregation, regulate mitochondrial function, and enhance autophagy, demonstrating their therapeutic potential for PD. Therefore, systematically reviewing the research status, hotspots, and trends of GLP-1 receptor agonists in PD is crucial for understanding the dynamic development of this field.

**Methods:**

Systematic searches were performed in the Web of Science Core Collection and Scopus databases to identify literature on GLP-1 receptor agonists and PD. Bibliometric analyses were conducted using CiteSpace and VOSviewer to evaluate publication trends, collaborative networks, co-citation patterns, and research hotspots. The search period spanned from database inception to April 14, 2026.

**Results:**

A total of 282 articles from WoS and Scopus were included for bibliometric analysis. The top three countries are China, the United States, and the United Kingdom. Major institutions include University College London, Shanxi Medical University, Henan University of Chinese Medicine, and Lancaster University, with authors Hölscher, Athauda, Aviles-Olmos, and Li. Hotspots focus on oxidative stress, insulin resistance, alpha-synuclein, blood–brain barrier, and exendin-4. Emerging themes with increasing research attention include mild cognitive impairment, GIP/GLP-1 dual agonists, and blood–brain barrier.

**Conclusion:**

PD is a multifactorial neurodegenerative disorder characterized by complex and heterogeneous pathological mechanisms, supporting the exploration of multi-target therapeutic strategies. GLP-1 receptor signaling is widely distributed in the central nervous system and peripheral metabolic tissues, suggesting a potential link between metabolic regulation and neurodegenerative processes. Bibliometric analysis indicates that GLP-1 receptor agonists have attracted increasing research attention in PD, particularly in relation to metabolic dysfunction, neuroinflammation, and mitochondrial pathways. However, current clinical evidence remains inconsistent, with findings influenced by drug heterogeneity, study populations, and methodological differences. Overall, GLP-1 receptor–related mechanisms represent an emerging and evolving research direction in PD, warranting further experimental and clinical investigation.

**Systematic review registration:**

https://osf.io/u7wdy

## 1 Preface

Parkinson’s disease (PD) is a common chronic neurodegenerative disease in middle-aged and elderly people, with the main pathological features being the progressive degeneration and loss of dopaminergic (DA) neurons in the substantia nigra of the midbrain and their axons, as well as the formation of Lewy bodies in the neuronal cytoplasm (Kalia and Lang, 2016). With the acceleration of population aging, PD has become one of the fastest-growing neurological diseases in the world. It is estimated that the total number of PD patients globally will increase to 25.2 million in 2050, a 112% increase compared with 2021, and the age-standardized prevalence will be 216 cases per 100,000, a 55% increase compared with 2021 (Su et al., 2025) which will bring a heavy disease burden to the world. With current medical capabilities, once diagnosed, PD patients cannot be cured, and current treatment still mainly focuses on controlling symptoms. Levodopa (L-DOPA) is the internationally recognized first-choice drug for controlling motor symptoms (Cajigal, 2021). However, as the disease progresses and DA neurons continue to degenerate, the clinical response to L-DOPA gradually fluctuates. Long-term use can lead to a decline in drug efficacy and induce motor complications such as motor fluctuations and dyskinesia, damaging patients’ motor function and quality of life. It is also one of the main factors causing disability in patients with advanced PD (Bjornestad et al., 2016; Riederer et al., 2025). Moreover, there is still a lack of long-term evidence indicating that L-DOPA can slow disease progression, and simple symptomatic treatment is difficult to meet the long-term management needs of PD. Exploring disease-modifying treatment is critical for achieving neuroprotection and delaying disease progression in PD patients.

In recent years, with the in-depth study of the pathological mechanism of PD, non-dopaminergic processes such as neuroinflammation, mitochondrial dysfunction, and metabolic disorders have been considered to play key roles in disease progression (Macedo et al., 2025). Among them, type 2 diabetes mellitus (T2DM) has been shown to significantly increase the risk of PD. Compared with the non-diabetic population, the risk of PD in T2DM patients increases by 27–32%. The risk is higher in patients with complex T2DM and those with early-onset disease, and longer disease duration and poorer glycemic control are also associated with higher risk (De Pablo-Fernandez et al., 2018; Aune et al., 2023). T2DM may contribute to the onset and progression of PD through mechanisms such as inducing mitochondrial dysfunction, exacerbating neuroinflammation, and promoting abnormal aggregation of α-synuclein, which provides a new direction for therapeutic strategies for PD (Hong et al., 2024). Preclinical studies have shown that GLP-1 receptor agonists, originally developed for the treatment of T2DM, may cross the blood–brain barrier to varying degrees and enter the central nervous system (CNS). They exert neuroprotective effects by inhibiting oxidative stress, reducing neuroinflammation, improving insulin resistance, and promoting neuroplasticity (Aviles-Olmos et al., 2025; Hölscher, 2025; Lee et al., 2025), thereby suggesting their potential as neuroprotective therapeutic candidates for PD. Although the neuroprotective mechanisms of GLP-1 receptor agonists in PD have been widely verified, clinical translation shows significant heterogeneity, and there are marked differences in pharmacodynamics and pharmacokinetics among drug classes and even among compounds within the same class (Zhang et al., 2025). Given the contradiction between the potential of GLP-1 receptor agonists in treating PD and the uncertainty of clinical evidence, it is essential to systematically review and summarize relevant studies.

Bibliometrics is a widely used approach for systematically analyzing research trends and status. CiteSpace (Chen, 2004) specializes in revealing knowledge structures and research frontiers through co-citation and burst detection; whereas VOSviewer (van Eck and Waltman, 2010) focuses on high-resolution co-occurrence network mapping. Together, they offer a comprehensive view of the evolving dynamics of a research field. To date, no bibliometric study has systematically examined the landscape of GLP-1 receptor agonists in PD. This study therefore uses CiteSpace and VOSviewer to perform visual analyses, construct knowledge maps, and identify research hotspots and emerging frontiers, aiming to provide insights into clinical heterogeneity and optimize therapeutic strategies. The study was pre-registered on OSF platform.^1^

## 2 Methods

### 2.1 Data sources and search strategies

Computer searches were conducted in the Web of Science Core Collection and Scopus databases to identify literature related to GLP-1 receptor agonists and PD. The search period covered database inception to April 14, 2026. The search strategy was developed collaboratively by the review team and reviewed by investigators experienced in systematic review methodology to ensure comprehensiveness and accuracy. The search strategy was based on free-text keywords, including disease names, drug names, synonyms, and relevant abbreviations. Using the Web of Science Core Collection as an example, the search strategy was as follows:(TS = (“Glucagon-Like Peptide-1 Receptor Agonists” OR “GLP-1 receptor agonist” OR “GLP-1 RA*” OR “GLP-1 analog*” OR “GLP-1 analog*” OR “GLP-1R agonist” OR “incretin mimetic” OR exenatide OR liraglutide OR lixisenatide OR albiglutide OR dulaglutide OR semaglutide OR tirzepatide OR retatrutide OR NLY01 OR Bydureon OR PT320 OR CagriSema OR Orforglipron OR Survodutide OR PB-119 OR DA5-CH OR DA-JC1 OR CT-996 OR Danuglipron OR Lotiglipton OR RGT-075 OR EXN1096)) AND TS = (“Parkinson’s disease” OR “parkinson*”). See the Supplementary materials for the complete search strategy.

### 2.2 Inclusion and exclusion criteria for literature

Inclusion and exclusion criteria were established according to the study objectives. Inclusion criteria were as follows: (1) published, peer-reviewed Articles or Reviews; (2) studies focusing on GLP-1 receptor agonists or investigational GLP-1-based therapies in PD, including clinical studies, preclinical studies, mechanistic investigations, and neuroprotection-related research; (3) publications written in English. Exclusion criteria were as follows: (1) conference abstracts, editorials, letters, news items, commentaries, corrections, book chapters, dissertations, preprints, gray literature, unpublished studies, ongoing clinical trials, and other non-peer-reviewed publications; (2) duplicate publications; (3) studies unrelated to the scope of this research or with unavailable full texts.

### 2.3 Literature screening and data standardization

The bibliographic information of the literature was imported into EndNote for duplicate checking. Two researchers independently screened the retrieved literature according to the above criteria. Disagreements were resolved through discussion. Finally, the screened literature was exported and saved in RefMan (RIS) format. Subsequently, CiteSpace was used to perform data normalization on the included literature: synonymous keywords were unified; for example, “glucagon-like peptide-1 receptor agonists” and “glp-1 receptor agonist” were merged into “GLP-1 receptor agonists.” Disease names were also standardized; for instance, “Parkinson disease” was merged into “Parkinson’s disease,” “Type 2 diabetes” into “type 2 diabetes mellitus,” and “Alzheimer’s disease” into “Alzheimer’s disease.” In addition, the country names of the affiliated authors were standardized. For example, “england,” “north ireland,” “scotland,” and “wales” were unified as “United Kingdom”; “USA” and “United States of America” were unified as “United States”; and “peoples r china” was unified as “China.”

### 2.4 Data analysis

The annual publication data were imported into Excel 2021 for analysis, and a line chart was generated. CiteSpace 6.4 R1 was used for knowledge graph analysis. The time span was set from 2002 to 2026, the time slice to 1 year, the g-index k-value to 25, the Top N parameter to 50, and the Top N% to 10%; the Pathfinder algorithm was used for pruning. Based on these parameters, the following networks were constructed: a national collaboration network, an institutional collaboration network, an author collaboration network, an author co-citation network, a journal dual-graph overlay, a reference co-citation network with its clustering map and timeline view, and a keyword co-occurrence network with its clustering map, timeline view, and burst detection. The nodes in the graphs represent analysis objects such as keywords, countries, institutions, or authors. The size of a node represents its frequency, and the color represents time or cluster membership; the connections between nodes represent associations such as co-occurrence, cooperation, or co-citation. Betweenness centrality is an important indicator for measuring the bridging role of a node in the network, and its calculation formula is as follows:


$$g\,\left(v\right)=\underset{s\neq v\neq t}{\sum }\frac{\sigma_{s\,t}\,\left(v\right)}{\sigma_{s\,t}}$$

Where σ_st_ is the total number of shortest paths from node s to t, and σ_st_(v) is the number of these paths passing through node v. Nodes with centrality ≥ 0.1 usually play a key connecting role between different research topics. Therefore, nodes with betweenness centrality ≥ 0.1 in the graphs generated by CiteSpace were marked with a purple outer circle to highlight their bridging role in knowledge flow. In addition, VOSviewer 1.6.20 was used for supplementary analysis; keyword co-occurrence analysis was performed to generate a density view, and the hotspot distribution was presented.

## 3 Results

**FIGURE 1:**
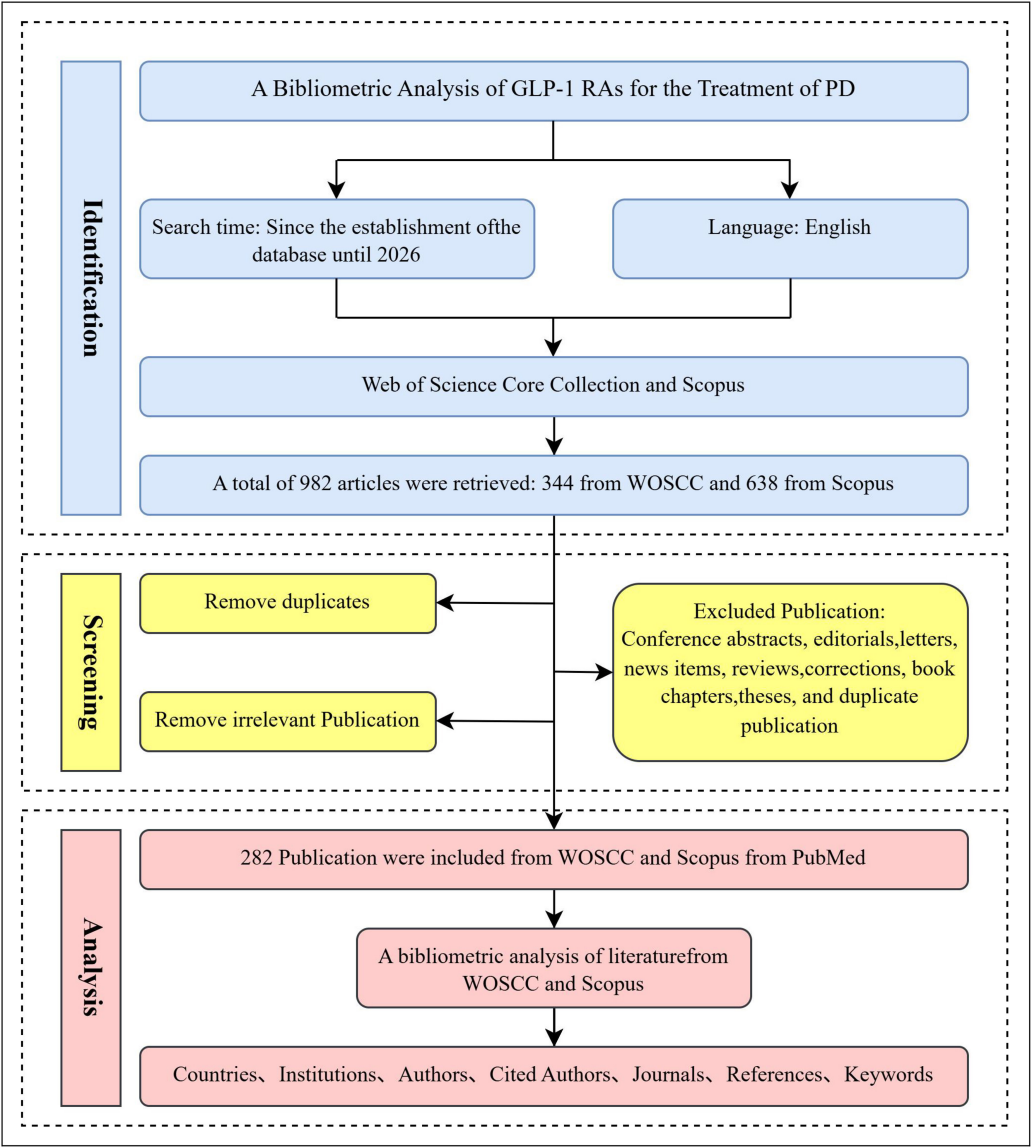
Flowchart of the literature screening process.

Our initial search of the Web of Science Core Collection and Scopus databases yielded a total of 982 articles; after deduplication and screening, 282 were ultimately included, as shown in Figure 1.

### 3.1 Literature publication volume

**FIGURE 2:**
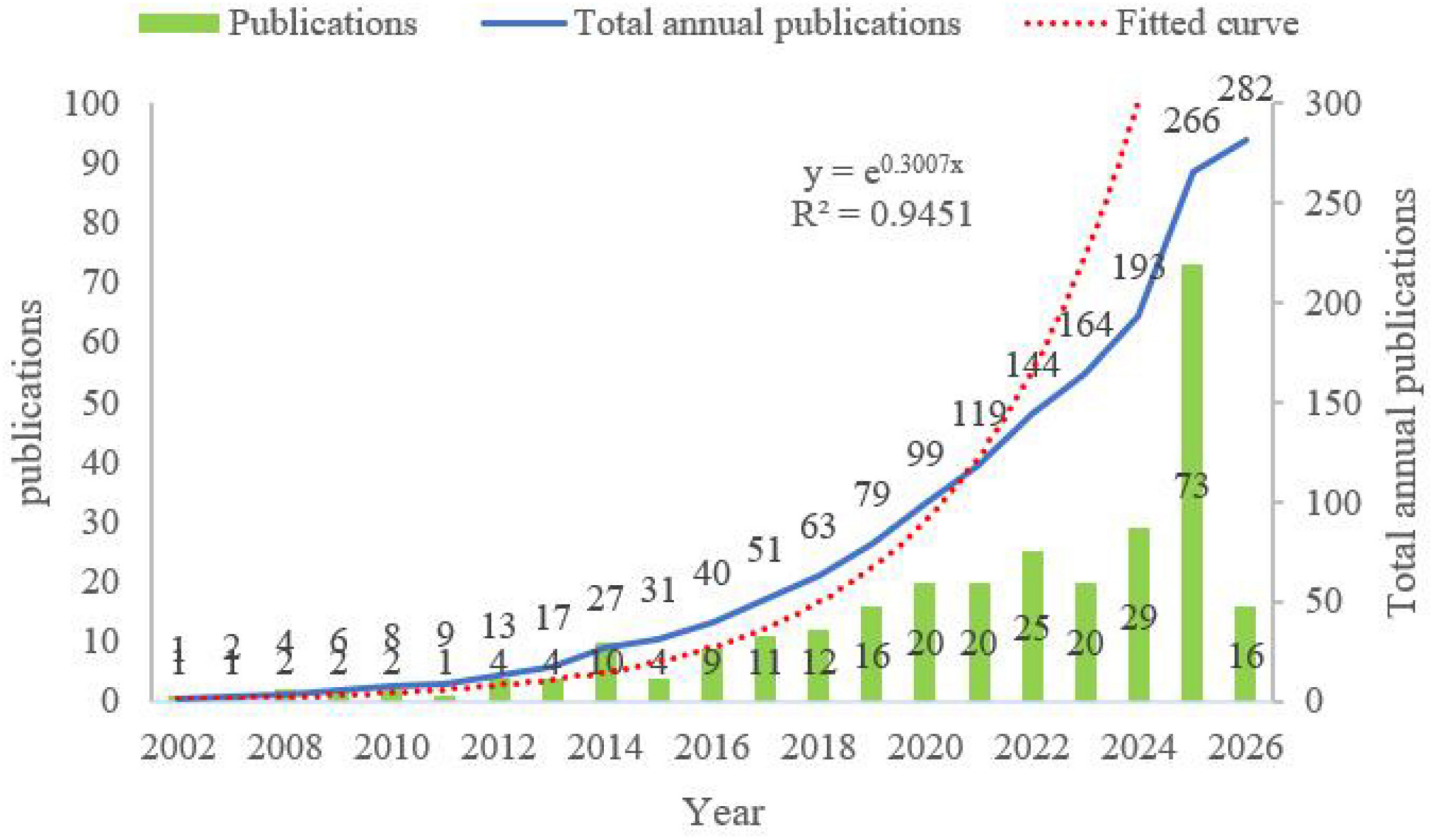
Trends in publication volume and cumulative publication volume of GLP-1 receptor agonists for the treatment of PD from 2002 to 2026.

Publication volume reflects the academic output and activity level of a research field within a given period, and serves as one of the most intuitive indicators of its development trends and shifts in research interest. A line chart was plotted with publication year on the horizontal axis and number of publications on the vertical axis (Figure 2). From 2002 to 2026, the annual publication volume on GLP-1 receptor agonists in PD showed a fluctuating upward trend, with a cumulative total of 282 publications. The period from 2002 to 2013 represented the initial stage, with an average of approximately 2 papers per year. Research during this phase mainly focused on the expression and distribution of GLP-1R in the central nervous system, as well as preliminary exploration of basic neuroprotective effects, such as anti-apoptotic activity and neurite outgrowth promotion. The period from 2014 to 2019 marked the early growth stage, averaging about 10 publications per year. During this phase, researchers began to investigate the epidemiological association between T2DM and PD and conducted numerous animal model studies to verify the mechanisms of GLP-1 receptor agonists, including anti-inflammatory, antioxidant, mitochondrial function improvement, and autophagy enhancement. From 2020 to 2026, the field entered a phase of rapid growth, with an average annual output of approximately 29 papers. The year 2025 was particularly notable, with 73 publications—a clear peak. However, as the literature search was conducted up to April 2026, the publication count for this year is incomplete and should be interpreted with caution; it is not directly comparable with full calendar years. Over this period, research has progressively shifted from preclinical investigations to clinical trials, with Phase I/II/III studies of GLP-1 receptor agonists for PD now underway, underscoring the growing recognition of this drug class as a core research hotspot for neuroprotective therapy.

### 3.2 National and institutional collaboration network

**FIGURE 3:**
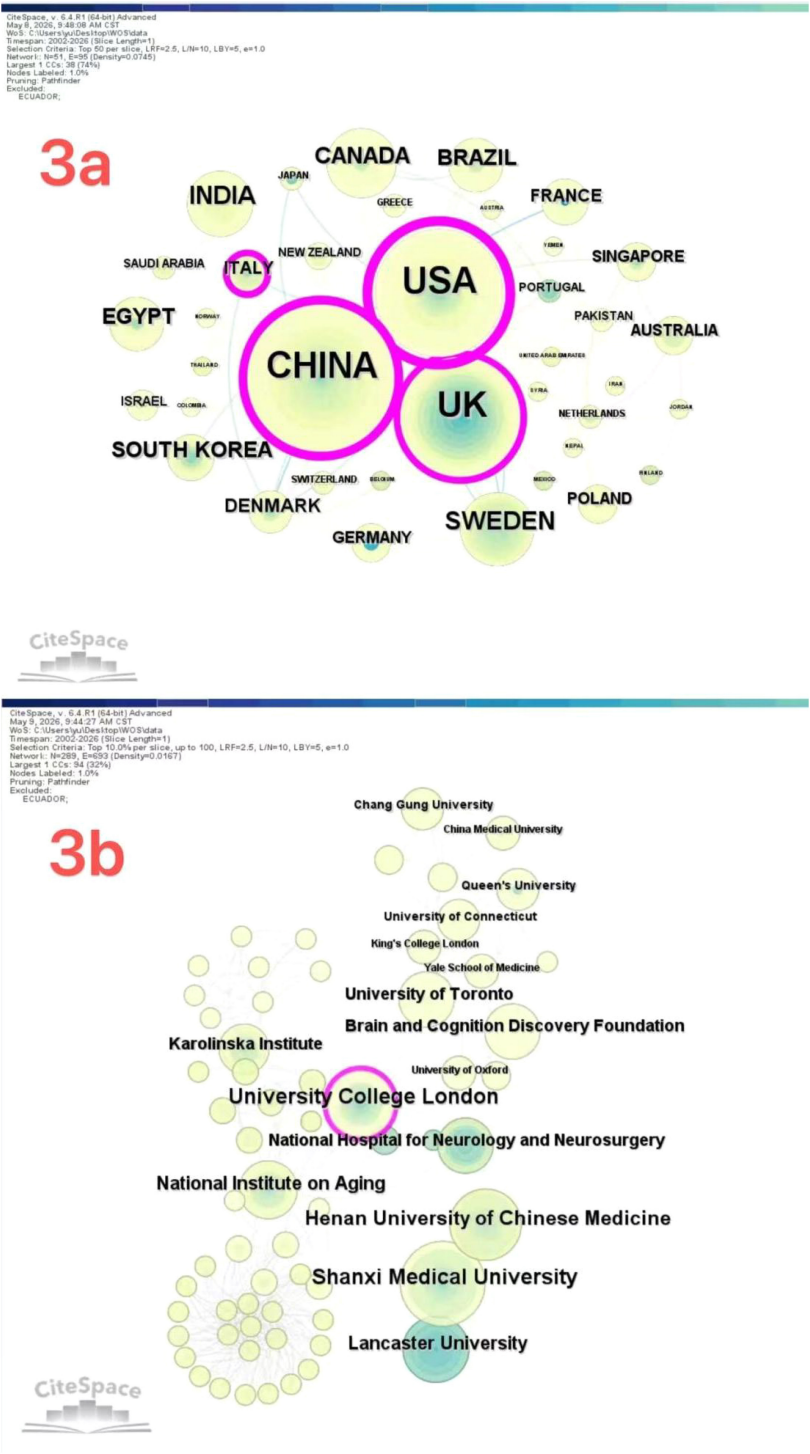
National and institutional cooperation network map. **(a)** Represents the national cooperation network and **(b)** represents the institutional cooperation network.

A collaboration network was constructed using “country” and “institution” as nodes to visualize collaboration patterns. The national collaboration network (Figure 3a) comprises 51 nodes (*N* = 51), 95 edges (*E* = 95), with a network density of 0.0745, indicating a moderate level of international collaboration in this field. The top 10 most productive countries are shown in Table 1. Among them, China, the United States, and the United Kingdom rank as the top three, contributing a total of 220 papers. All three countries are marked with a purple outer ring (betweenness centrality ≥ 0.1), indicating that they serve not only as major contributors but also as key bridges within the collaboration network. Additionally, although Italy does not rank among the top ten in publication volume, its relatively high betweenness centrality suggests a pivotal connecting role in European and cross-regional collaborations. The institutional collaboration network (Figure 3b) includes 289 nodes (*N* = 289) and 693 edges (*E* = 693), with a network density of 0.0167, suggesting relatively dispersed collaborative relationships among institutions, with major research centers spanning Asia, Europe, and North America. The top 10 institutions are listed in Table 1. University College London (UCL) ranks first with 17 publications and is marked with a purple outer ring (betweenness centrality ≥ 0.1), indicating that UCL is not only the most productive and influential institution but also serves as a hub connecting multiple research centers. Shanxi Medical University and Henan University of Chinese Medicine rank second and third, reflecting the strong research activity and growing influence of Chinese institutions in this field.

**TABLE 1:** Top 10 most productive countries and institutions.

Rank	Country	Count	Citations	Rank	Institution	Count	Citations
1	China	82	4,483	1	University College London	17	1,325
2	United States	71	5,132	2	Shanxi Medical University	16	2,302
3	United Kingdom	67	7,014	3	Henan University of Chinese Medicine	13	1,208
4	Sweden	19	482	4	Lancaster University	12	2,211
5	India	16	305	5	National Institute on Aging	11	2367
6	Canada	15	542	6	University of Toronto	8	345
7	Brazil	12	394	7	Karolinska Institute	8	441
8	Egypt	12	514	8	Brain and Cognition Discovery Foundation	7	58
9	South Korea	10	1,450	9	National Hospital for Neurology and Neurosurgery	7	1,339
10	Denmark	9	494	10	Chang Gung University	4	136

### 3.3 Analysis of author co-citation network and author collaboration network

**FIGURE 4:**
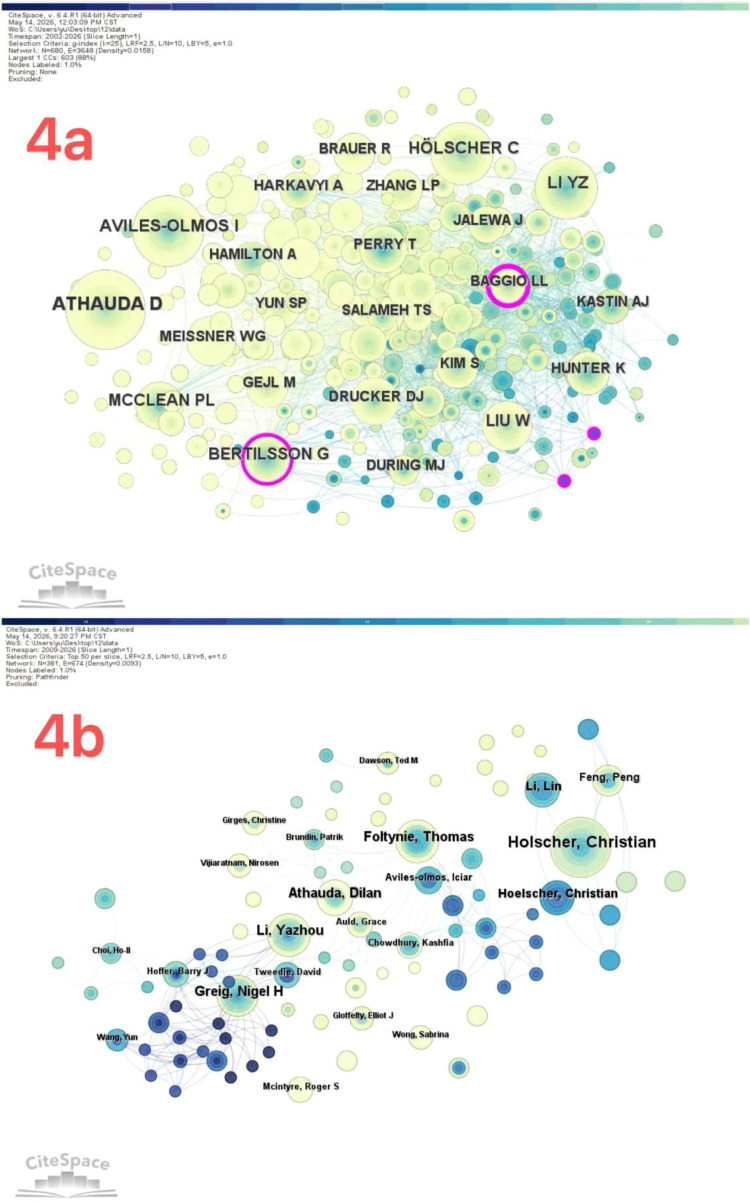
Author co-citation network and author collaboration network map. **(a)** Shows the author co-citation network and **(b)** shows the author collaboration network.

To identify influential authors in the field of GLP-1 receptor agonists for PD treatment, CiteSpace was used to construct author co-citation and collaboration network maps (Figure 4). The author co-citation network (Figure 4a) comprises 680 nodes (*N* = 680) and 3,648 edges (*E* = 3,648), with a network density of 0.0158; the largest connected component accounts for 88% of all nodes, indicating that the knowledge base in this field is highly integrated and that most highly cited scholars are closely linked through co-citation relationships. The top 10 most cited authors are shown in Table 2. Athauda, Aviles-Olmos, Li, Hölscher, and Liu rank as the top five, jointly constituting the core knowledge framework of this field. Beyond citation counts, Bertilsson and Baggio exhibit high betweenness centrality. Bertilsson’s early study demonstrated that exendin-4 promotes adult neurogenesis *in vitro* and *in vivo*, corrects dopamine imbalance, increases the number of tyrosine hydroxylase-positive dopaminergic neurons in the substantia nigra of PD rat models, and improves motor function (Bertilsson et al., 2008). Baggio’s work has explored the expression and function of GLP-1 receptors in the central nervous system (Baggio and Drucker, 2014) . These two researchers serve as important bridges between basic mechanistic research and clinical translation. The author collaboration network (Figure 4b) includes 381 nodes (*N* = 381) and 674 edges (*E* = 674), with a network density of 0.0093, indicating that direct co-authorship among researchers is relatively sparse. The top 10 most productive authors are listed in Table 2. Hölscher (33 papers) ranks first as the most active researcher in this field, followed by Foltynie (18 papers) and Greig (16 papers). Notably, core scholars such as Hölscher, Athauda, Aviles-Olmos, and Li appear prominently in both the co-citation and collaboration networks, indicating that these researchers are not only foundational contributors to the knowledge base but also current leaders driving research activities in this field.

**TABLE 2:** Top 10 authors by citation count and publication count.

Rank	Cited author	Count	Year	Author	Count	Year
1	Dilan Athauda	196	2017	Christian Hölscher	33	2016
2	Iciar Aviles-Olmos	167	2012	Tom Foltynie	18	2009
3	Yazhou Li	142	2009	Nigel H. Greig	16	2013
4	Christian Hölscher	139	2012	Dilan Athauda	13	2009
5	Weizhen Liu	97	2015	Liang Yu	7	2016
6	Göran Bertilsson	92	2009	Iciar Aviles-Olmos	7	2012
7	Paula L. McClean	92	2012	Yazhou Li	7	2015
8	Sehee Kim	90	2010	Roger S. McIntyre	7	2016
9	Alexander Harkavyi	88	2009	Peng Feng	7	2013
10	TracyAnn Perry	79	2009	Patrik Brundin	5	2009

### 3.4 Journal double graph overlay

**FIGURE 5:**
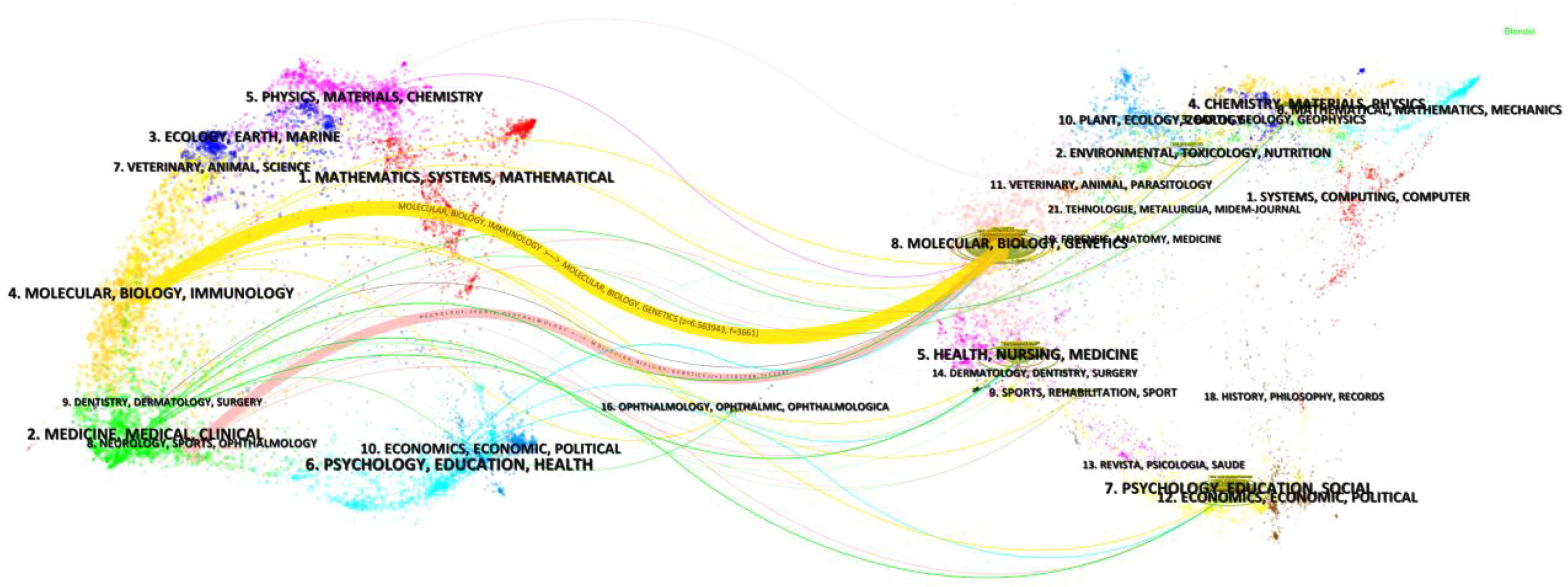
Overlay graph of the journal’s double graphs.

The overlay graph of the journal’s double graphs shows the positioning of the research topic in the disciplinary system, tracks the knowledge citation relationships at the journal level, and reflects the dynamic evolution of the research hotspots in this field. As shown in Figure 5, the journals on the left are the citing journals, and the journals on the right are the cited journals. The curve between them represents the citation path. As shown, the core knowledge base of the research on the treatment of PD with GLP-1 receptor agonists comes from the “MOLECULAR BIOLOGY, GENETICS” cluster on the right and migrates to the “MOLECULAR BIOLOGY, IMMUNOLOGY” and “NEUROLOGY, SPORTS, OPHTHALMOLOGY” clusters on the left. This indicates that this field is based on molecular biology and genetics and extends to the application of immune mechanisms and neuroscience, reflecting the interdisciplinary knowledge integration from basic mechanisms to clinical neuroprotection.

### 3.5 References analysis

#### 3.5.1 Co-citation network and clustering map

**FIGURE 6:**
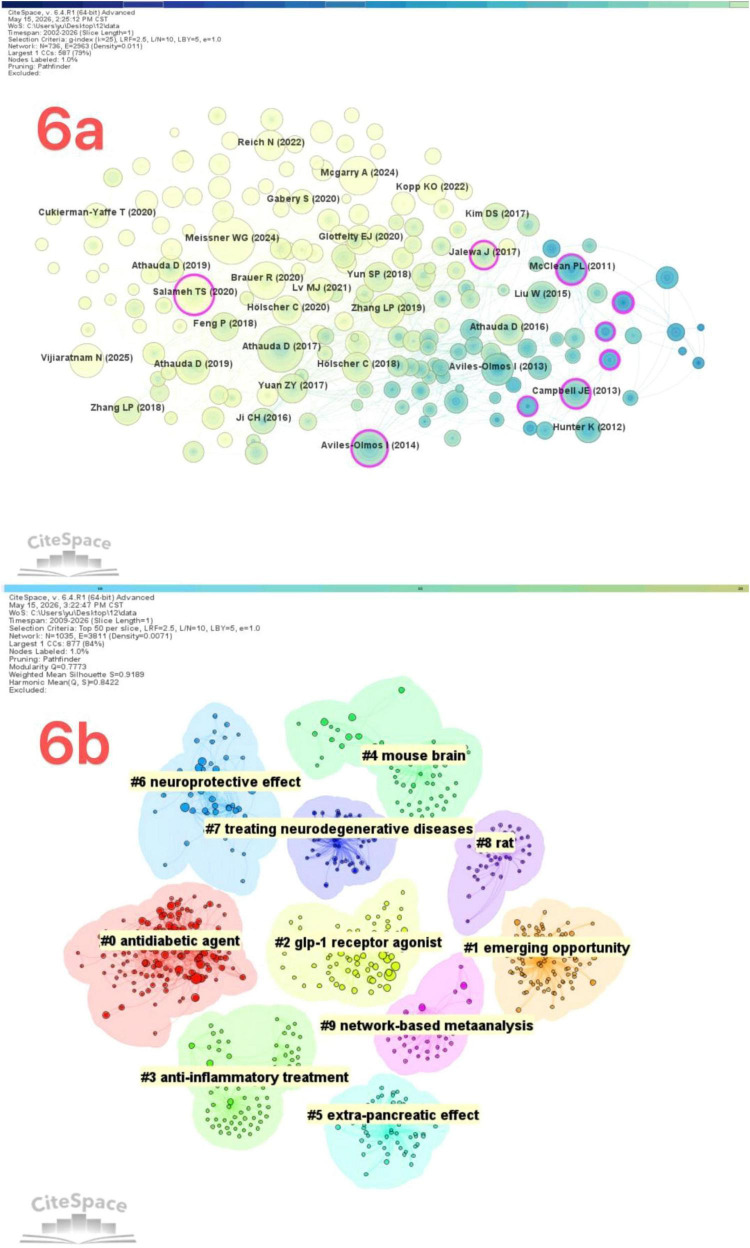
Co-citation network and clustering map of references. **(a)** Shows the co-citation network of references and **(b)** shows the clustering map of references.

Co-citation analysis of references was performed to identify the knowledge base in the field of GLP-1 receptor agonists for PD treatment. A co-citation network was constructed using CiteSpace (Figure 6a). This network comprises 736 nodes (*N* = 736) and 2,963 edges (*E* = 2,963), with a network density of 0.011; the largest connected component accounts for 79% of all nodes, indicating that the core literature in this field is relatively concentrated and that most highly cited publications are interconnected through co-citation relationships. Based on this network, cluster analysis was performed using the Log-Likelihood Ratio (LLR) algorithm to extract cluster labels, resulting in 10 distinct clusters (Figure 6b). The quality of the clustering results can be evaluated using the modularity value (Q) and the average silhouette value (S). A *Q* > 0.3 indicates a statistically meaningful clustering structure, while an S value ≥ 0.7 suggests good internal consistency. Here, the Q value is 0.7773 and the S value is 0.9189, indicating that the clustering structure is robust and internally homogeneous. Antidiabetic agent (#0), as the largest core cluster, suggests that GLP-1 receptor agonists initially entered the research field as antidiabetic drugs, revealing the epidemiological link between T2DM and PD. The GLP-1 receptor agonist (#2) represents the drug class under investigation, with its core target being GLP-1R. The extra-pancreatic effect (#5) and the neuroprotective effect (#6) jointly illustrate the extra-pancreatic effects of GLP-1 receptor agonists beyond their antidiabetic properties and their disease-modifying potential in the central nervous system. Anti-inflammatory treatment (#3) further highlights that the anti-inflammatory effect is one of the pleiotropic neuroprotective mechanisms of GLP-1 receptor agonists, in addition to their antidiabetic effects (Diz-Chaves et al., 2022). Mouse brain (#4) and rat (#8) reflect the preclinical research evidence in this field, providing critical support for subsequent clinical translation. Network-based meta-analysis (#9) indicates that the field has progressed toward integrating high-level evidence, while treating neurodegenerative diseases (#7) expands the research scope to other neurodegenerative diseases such as AD and HD. Finally, emerging opportunity (#1) reflects the growing prospects for repurposing GLP-1 receptor agonists from the antidiabetic field to neuroprotective treatments such as PD.

#### 3.5.2 Timeline view

**FIGURE 7:**
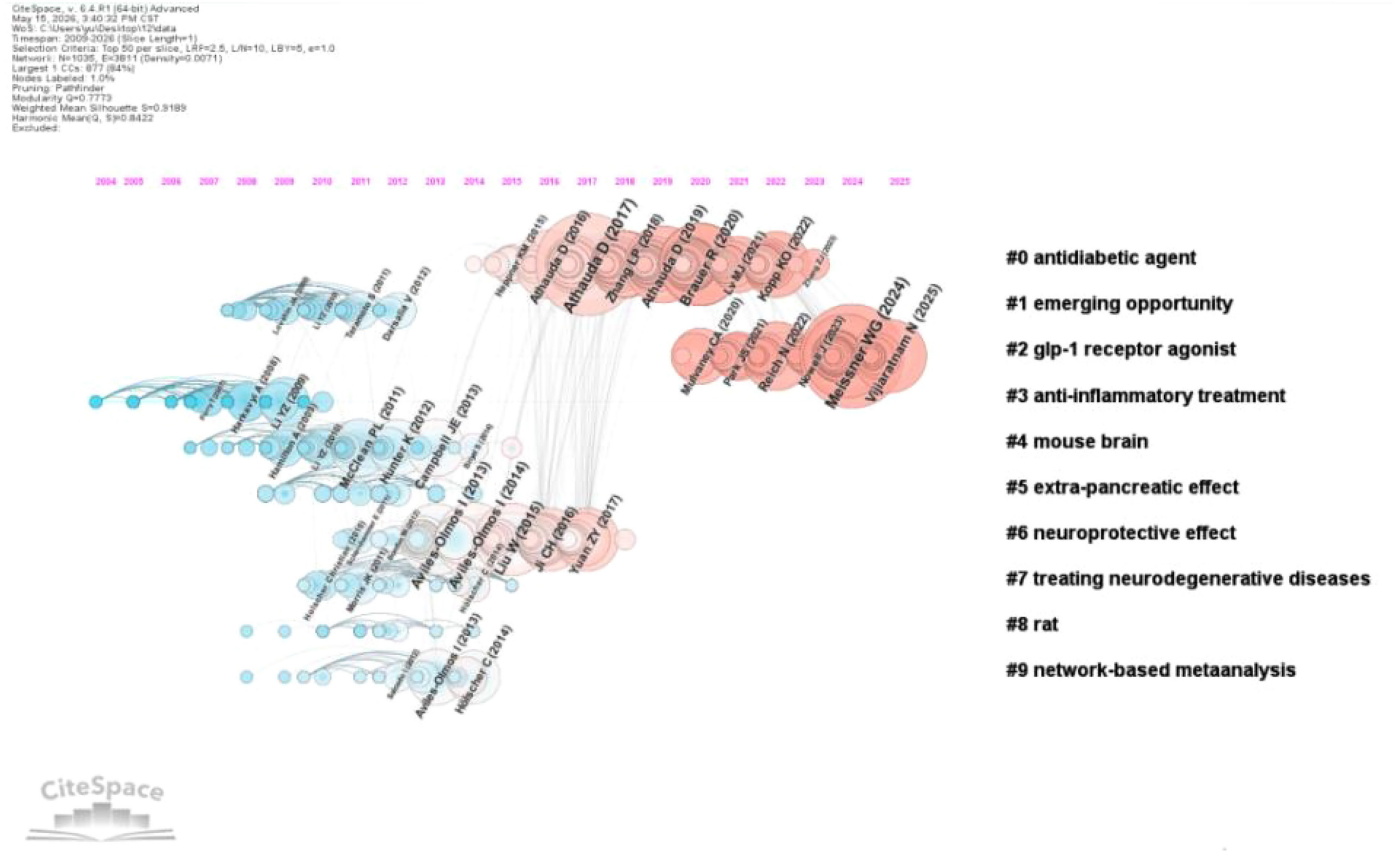
Co-citation cluster timeline map of references.

The timeline view can effectively display the temporal evolution pattern and research trends of cited documents in different research clusters. The timeline view (Figure 7) was generated based on the co-cited document clustering results. As shown in the figure, early research in this field focused largely on the extrapancreatic effects and neuroprotective mechanisms of GLP-1 receptor agonists. Numerous studies concentrated on rodent models, systematically elucidating core pathways that mediate neuroprotection, such as anti-inflammation, mitochondrial protection, enhanced autophagy, and inhibition of protein aggregation (Wang et al., 2024; Lee et al., 2025; Sioufi et al., 2026). A key milestone was the 2013 open-label proof-of-concept trial of exenatide by Aviles-Olmos et al. (2013), which first introduced GLP-1 receptor agonists into human PD research and shifted the field from animal studies toward clinical feasibility exploration. Subsequently, Athauda et al. (2017) further verified its potential disease-modifying effect in the first randomized double-blind placebo-controlled trial, which was completed in 2017 and laid a preliminary foundation for the therapeutic framework of GLP-1 receptor agonists in neurodegenerative diseases. In recent years, with the successive publication of multiple randomized controlled trial results, this field has entered a phase of accumulating clinical evidence. However, the currently published trials remain controversial, and the overall evidence is limited in strength. Consequently, research focus has shifted toward systematic reviews and meta-analyses, which aim to clarify efficacy and explore sources of heterogeneity by integrating high-level evidence. Overall, research on GLP-1 receptor agonists for PD has evolved from pathological observations in animal models to clinical translation exploration, and from early feasibility verification to evidence-based assessment, in an effort to determine whether these agents hold disease-modifying potential.

#### 3.5.3 Highly co-cited representative documents

Citation frequency is an important indicator of a publication’s contribution to the field. The top 10 most cited references are shown in Table 3. These publications cover the complete evidence chain, spanning epidemiology, preclinical mechanisms, and clinical trials. Nine of them were published in Q1 journals, including three in The Lancet and its subsidiaries, one in the New England Journal of Medicine, and six in leading general medicine and neuroscience journals such as JAMA Neurology and Brain, reflecting the high academic influence and level of evidence of research in this field. Among the preclinical and population-level studies, a prospective cohort study by Brauer et al. (2020) based on the UK’s Health Improvement Network database, which included 100,288 patients with type 2 diabetes, demonstrated that the risk of PD was significantly reduced in patients taking GLP-1 receptor agonists, providing epidemiological support for a drug repurposing strategy. Salameh et al. (2020) revealed substantial inter-drug differences in blood-brain barrier penetration among incretin receptor agonists through pharmacokinetic studies, showing that unacylated and unPEGylated agents enter the brain primarily via adsorptive-mediated transcytosis, thus addressing a critical gap in quantitative research on the central bioavailability of GLP-1 drugs. Zhang et al. (2019) and Liu et al. (2015) confirmed the neuroprotective effects of semaglutide, lixisenatide, and liraglutide in MPTP mouse models, demonstrating reductions in α-synuclein levels and improvements in motor function, and elucidating core mechanisms such as anti-inflammatory activity and regulation of protein aggregation. In clinical trials, the most cited one is the exenatide randomized double-blind placebo-controlled trial by Athauda et al. (2017) published in The Lancet, which first confirmed that GLP-1 receptor agonists can improve the motor and cognitive functions of early PD patients; the LIXIPARK phase II clinical trial by Meissner et al. (2024), published in the New England Journal of Medicine, further showed that lixisenatide inhibited the deterioration of motor function in early PD, providing the strongest clinical signal to date for a potential disease-modifying effect. However, the exenatide phase III randomized controlled trial by Vijiaratnam et al. (2025), published in The Lancet, although confirming the safety and tolerability of the drug, did not significantly slow disease progression, representing the highest-level negative clinical evidence currently available. The coexistence of these positive and negative results highlights the controversial clinical efficacy of GLP-1 receptor agonists in PD treatment and underscores that evidence heterogeneity across different drugs and trial designs remains the core challenge in this field.

**TABLE 3:** Top 10 references ranked by co-citation frequency.

Rank	Cited frequency	Title	First author	Journal	Year
1	71	Exenatide once weekly versus placebo in Parkinson’s disease: a randomized, double-blind, placebo-controlled trial	Dilan Athauda	The Lancet (88.5, Q1)	2017
2	70	Trial of lixisenatide in early Parkinson’s disease	Wassilios G. Meissner	New England journal of medicine (78.5, Q1)	2024
3	47	Safety, tolerability, and efficacy of NLY01 in early untreated Parkinson’s disease: a randomized, double-blind, placebo-controlled trial	Andrew McGarry	Lancet neurology (45.5, Q1)	2024
4	46	Diabetes medications and risk of Parkinson’s disease: a cohort study of patients with diabetes	Ruth Brauer	Brain (11.7, Q1)	2020
5	46	Brain uptake pharmacokinetics of incretin receptor agonists showing promise as Alzheimer’s and Parkinson’s disease therapeutics	Therese S. Salameh	Biochemical pharmacology (5.6, Q1)	2020
6	39	Motor and cognitive advantages persist 12 months after exenatide exposure in Parkinson’s disease	Iciar Aviles-Olmos	Journal of Parkinson’s disease (5.0, Q1)	2014
7	39	Utility of neuronal-derived exosomes to examine molecular mechanisms that affect motor function in patients with Parkinson disease a secondary analysis of the exenatide-PD trial	Dilan Athauda	JAMA neurology (21.4, Q1)	2019
8	39	Semaglutide is neuroprotective and reduces α-synuclein levels in the chronic MPTP mouse model of Parkinson’s disease.	Liping Zhang	Journal of Parkinson’s disease (5.0, Q1)	2019
9	36	Neuroprotective effects of lixisenatide and liraglutide in the 1-methyl-4-phenyl-1,2,3,6-tetrahydropyridine mouse model of Parkinson’s disease	Weizhen Liu	Neuroscience (2.8, Q2)	2015
10	35	Exenatide once a week versus placebo as apotential disease- modifying treatment for people with Parkinson’s disease in the United Kingdom: a phase 3, multicentre, double-blind, parallel-group, randomized, placebo-controlled trial	Nirosen Vijiaratnam	The Lancet (88.5, Q1)	2025

### 3.6 Keyword analysis

#### 3.6.1 Keyword co-occurrence network and density view

**FIGURE 8:**
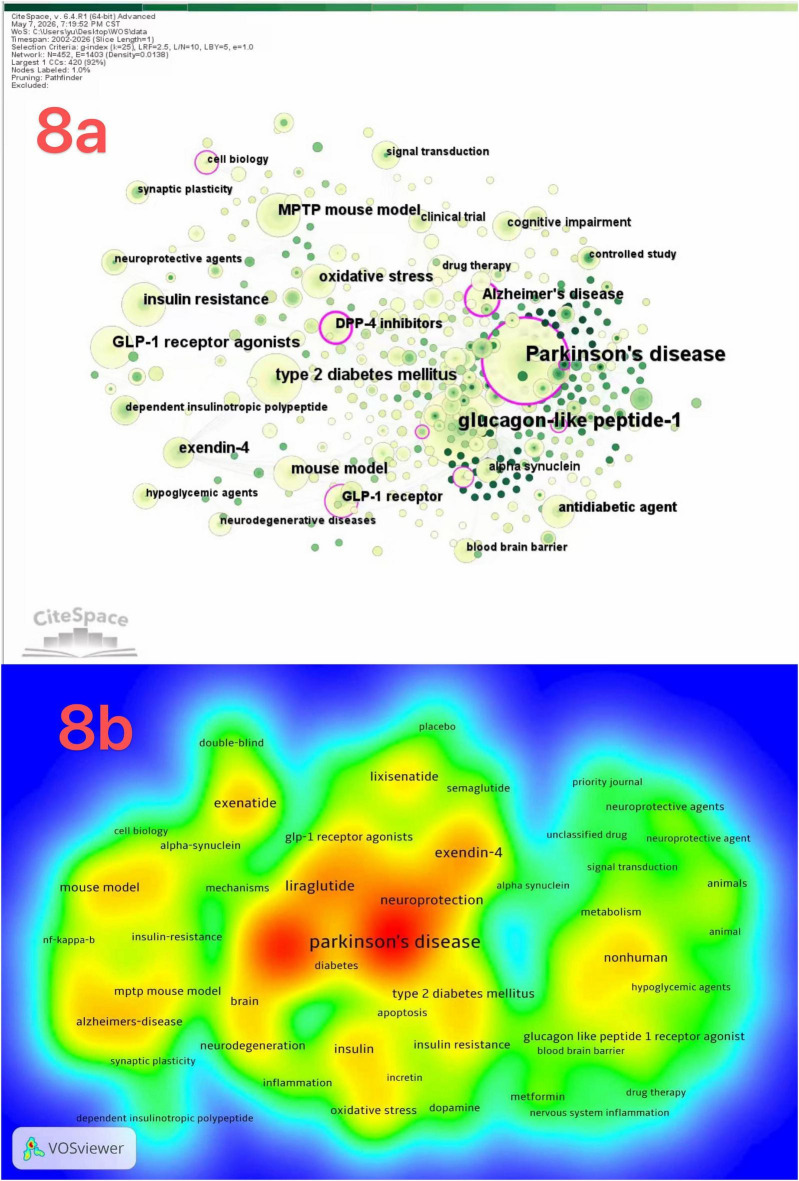
Keyword co-occurrence network and density view. **(a)** Shows the keyword co-occurrence network and **(b)** shows the keyword density view.

Keyword co-occurrence network analysis can reveal hot topics, knowledge structure, and evolutionary context in a research field. A keyword co-occurrence network was constructed using CiteSpace for the field of GLP-1 receptor agonists in PD treatment (Figure 8a). This network comprises 452 nodes (*N* = 452) and 1,403 edges (*E* = 1,403), with a network density of 0.0138; the largest connected component accounts for 92% of all nodes, indicating that keyword co-occurrence relationships are highly concentrated and that core research topics form a tightly interconnected network. The top 15 high-frequency keywords are shown in Table 4 and can be classified into four major themes: disease targets, drugs and targets, mechanisms and models, and clinical and disciplinary backgrounds.

**TABLE 4:** Top 15 high-frequency keywords in research on GLP-1 receptor agonists for PD.

Rank	Keyword	Count	Centrality	Year
1	Parkinson’s disease	183	0.28	2002
2	Glucagon-like peptide-1	139	0.07	2002
3	Type 2 diabetes mellitus	64	0.05	2014
4	GLP-1 receptor agonists	49	0.02	2014
5	Mouse model	44	0.09	2012
6	Exendin-4	42	0.07	2002
7	Oxidative stress	40	0.04	2004
8	Alzheimer’s disease	39	0.25	2002
9	MPTP mouse model	36	0.06	2016
10	Insulin resistance	36	0.02	2016
11	GLP-1 receptor	25	0.19	2004
12	DPP-4 inhibitors	24	0.20	2014
13	Antidiabetic agent	24	0.06	2009
14	Cognitive impairment	22	0.00	2016
15	Clinical trial	17	0.07	2013

In the disease target theme, “Parkinson’s disease” has the highest frequency and the highest betweenness centrality (0.28), making it a hub of the knowledge network in this field. “Alzheimer’s disease” also has a relatively high betweenness centrality (0.25), indicating that research on the neuroprotective effects of GLP-1 receptor agonists has attracted attention in other neurodegenerative diseases, such as AD. “Cognitive impairment” suggests that the potential of GLP-1 receptor agonists to improve cognitive impairment related to PD and other neurodegenerative diseases is one of the important directions of current research. In the drug and target theme, high-frequency keywords such as “glucagon-like peptide-1,” “GLP-1 receptor agonists,” “GLP-1 receptor,” “exendin-4,” and “antidiabetic agent” indicate that the GLP-1 signaling axis and its receptor agonists hold significant promise in the disease-modifying treatment of PD, with “exendin-4” having been widely used in preclinical and clinical research in this field. In addition, “GLP-1 receptor” has a relatively high betweenness centrality (0.19), suggesting that this receptor plays a key bridging role in connecting drug actions to downstream signaling pathways. The mechanisms and models theme covers two core mechanisms, namely “oxidative stress” and “insulin resistance,” as well as preclinical experimental systems such as “mouse model” and “MPTP mouse model.” As a core link in PD pathogenesis, “oxidative stress” reflects the vicious cycle in which mitochondrial dysfunction and dopamine oxidative damage exacerbate each other, both of which play key roles in PD (Chakrabarti and Bisaglia, 2023). The role of “insulin resistance” in PD pathogenesis has been extensively explored; studies have shown that impaired brain insulin signaling pathways contribute to PD pathology by promoting α-synuclein aggregation, inducing mitochondrial dysfunction, and exacerbating neuroinflammation (Hölscher, 2020). In the clinical and disciplinary background theme, “type 2 diabetes mellitus” provides the disciplinary foundation for the use of GLP-1 receptor agonists in PD treatment. “DPP-4 inhibitors,” as a keyword with high betweenness centrality (0.20), serves as a key bridge connecting research on endocrine metabolism and neurodegenerative diseases, suggesting that cross-drug comparisons within the same incretin pathway represent an important avenue for knowledge expansion in this field. Finally, “clinical trial” marks the transition of this field from mechanistic research to evidence-based clinical validation.

To further supplement the knowledge structure of this field from the perspective of heat distribution, a keyword density view was generated based on VOSviewer (Figure 8b). This figure presents the spatial aggregation intensity of knowledge hotspots using a color gradient, where bright yellow and red areas indicate highly concentrated research hotspots. In addition to the topic terms “Parkinson’s disease” and “GLP-1 receptor agonists,” a high-density hot zone composed of “exenatide,” “liraglutide,” “lixisenatide,” and “semaglutide” forms in the center and upper-middle region of the density view, suggesting that the field has expanded from early single-drug studies to comparisons of multi-generational GLP-1 receptor agonists. Surrounding this hot zone, “double-blind” and “placebo” cluster with medium-to-high heat, indicating that randomized double-blind placebo-controlled trials are being increasingly used to validate the potential efficacy of this drug class in treating PD. Moreover, the low-to-medium heat distribution of “blood–brain barrier” in the peripheral area suggests that the central delivery bottleneck remains a key scientific issue that remains to be addressed but has not yet become a research hotspot in this field.

#### 3.6.2 Keyword clustering map and timeline view

**FIGURE 9:**
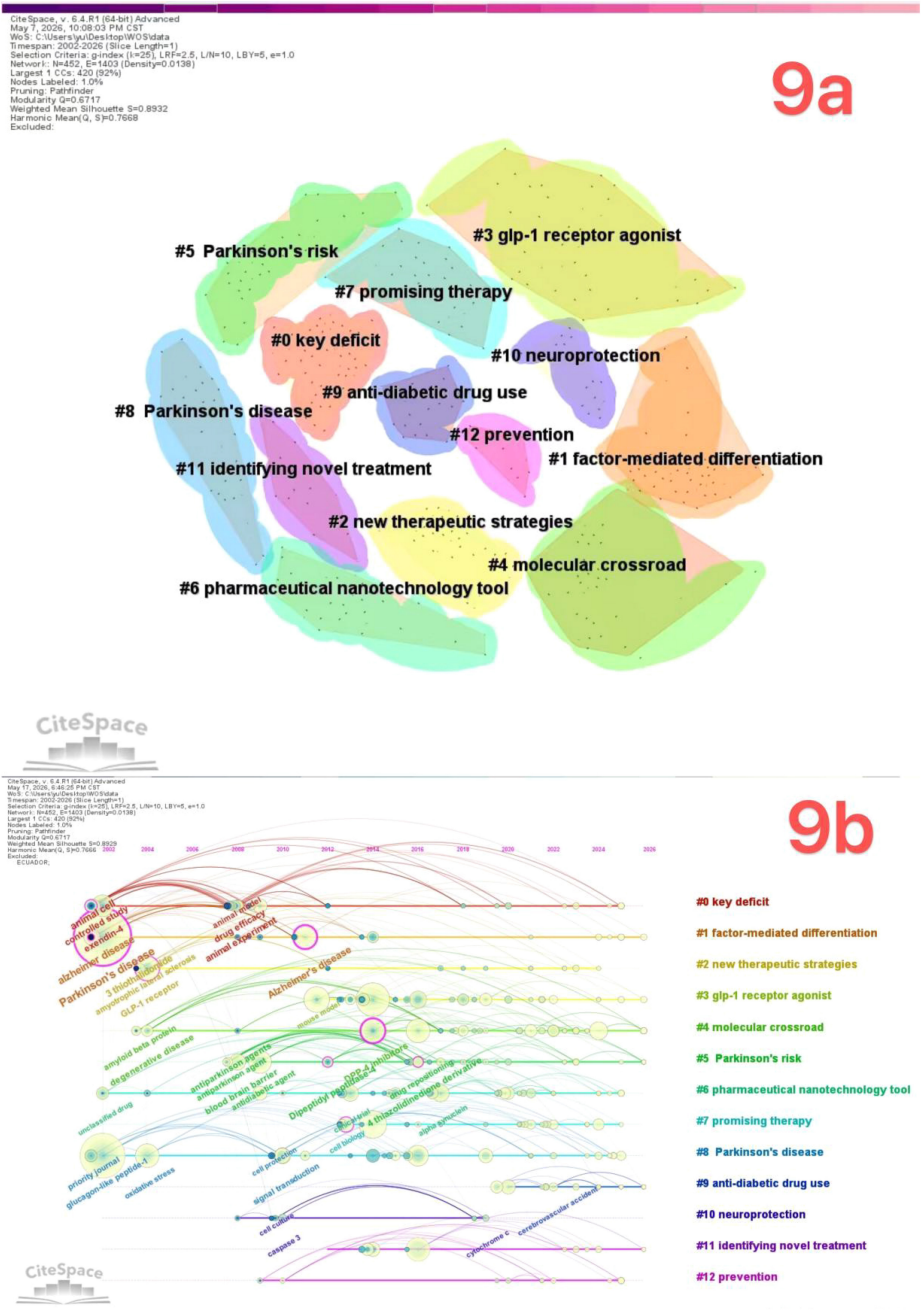
Keyword clustering map and timeline view. **(a)** Shows the keyword clustering map and **(b)** shows the keyword timeline view.

Keyword clustering analysis can identify the modular features and thematic associations of the knowledge structure in this field. Based on the keyword co-occurrence network, the LLR algorithm was used to extract clustering labels, forming a total of 13 main clusters (Figure 9a). The Q value is 0.6717 ( > 0.3) and the S value is 0.8932 ( > 0.7), indicating a robust clustering structure with good internal homogeneity and well-defined boundaries between clusters. These 13 clusters can be summarized into four major themes: disease pathology, drug repurposing and disciplinary origins, mechanisms and pathways, and treatment translation and technology. The disease and pathological basis theme has Parkinson’s disease (#8) as its core. Parkinson’s risk (#5) focuses on epidemiological risk associations, key deficit (#0) points to core pathological abnormalities such as dopaminergic neuron degeneration, and prevention (#12) reflects the shift of research focus from symptomatic treatment to early prevention, forming an overall research pattern from risk identification and defect clarification to prevention exploration. The drugs and disciplinary origins theme includes GLP-1 receptor agonist (#3) and anti-diabetic drug use (#9), intuitively presenting the “new use of old drugs” disciplinary feature of GLP-1 receptor agonists spanning from diabetes treatment to neurodegenerative diseases. The mechanisms and pathways theme covers neuroprotection (#10), molecular crossroad (#4), and factor-mediated differentiation (#1). Neuroprotection (#10) connects drug intervention and disease targets through its neuroprotective mechanisms, serving as a key hub for translating mechanistic research into clinical practice; molecular crossroad (#4) represents the intersection and integration point of multiple signaling pathways; factor-mediated differentiation (#1) may point to emerging frontier directions such as nerve regeneration and stem cell-directed differentiation. The treatment translation and technology module includes promising therapy (#7), new therapeutic strategies (#2), identifying novel treatment (#11), and pharmaceutical nanotechnology tool (#6). This module collectively reflects the diverse explorations at the clinical translation level. Among them, promising therapy (#7) highlights the pivotal role of clinical translation from risk identification to drug intervention; pharmaceutical nanotechnology tool (#6) suggests that drug delivery technologies—such as enhancing blood–brain barrier penetration—represent an emerging theme for improving the central efficacy of GLP-1 receptor agonists. The keyword timeline view (Figure 9b) clearly shows the research hotspots and evolution trends in this field.

#### 3.6.3 Keyword emergence detection

**FIGURE 10:**
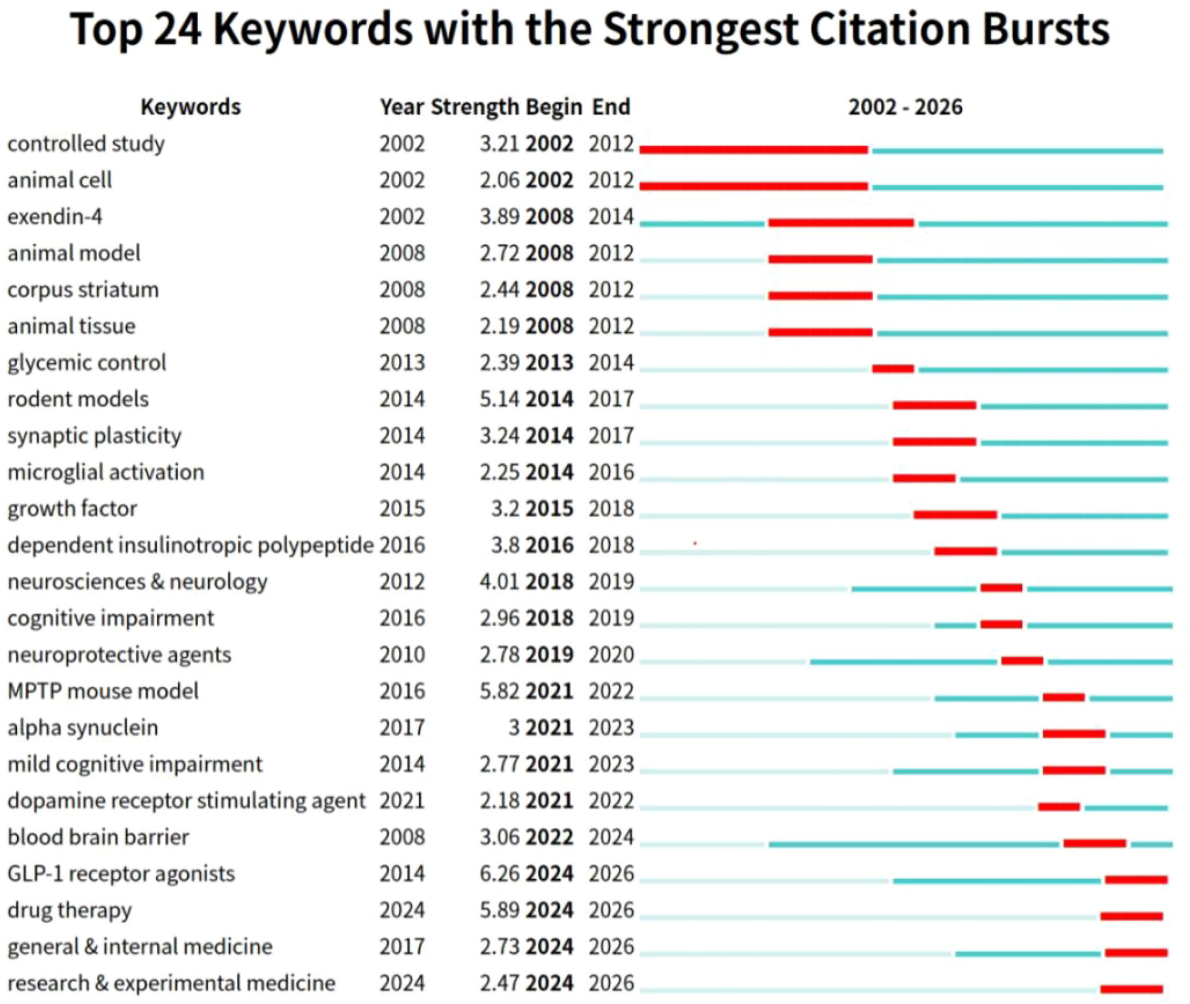
Keyword timeline view and emergence map.

Emergence detection can identify keywords with a sudden increase in citation heat during a certain period. As shown in Figure 10, the emergence of “general and internal medicine” and “research and experimental medicine” (2024–2026) reflects the diffusion of academic influence in this field from specialized journals to comprehensive medical journals, indicating that research results are receiving more extensive interdisciplinary attention. The emergence intensity of “GLP-1 receptor agonists” (Strength 6.26) and “drug therapy” (Strength 5.89) is the highest, suggesting that drug repositioning and clinical translation research are in a period of rapid growth, indicating that the potential disease-modifying therapy of GLP-1 receptor agonists is receiving high attention. The emergence words such as “blood brain barrier” (Strength 3.06), “alpha synuclein” (Strength 3), and “mild cognitive impairment” (Strength 2.77) reflect the continuous attention to directions such as drug delivery, protein pathology, and cognitive impairment, and represent emerging themes with increasing research interest.

## 4 Discussion

### 4.1 Overall development trend

This study provides a comprehensive bibliometric overview of GLP-1 receptor agonists in PD research. The growing publication output, particularly after 2020, indicates a progressive shift from preclinical exploration toward clinical translation. At the country and institutional levels, the leading contributions from China, the United States, and the United Kingdom, together with key hubs such as University College London, suggest well-established international collaborative networks. The high productivity and citation impact of core authors such as Hölscher, Athauda, and Li further confirm the presence of an influential research community. The concentration of highly cited references in high-impact journals reflects the broad academic recognition and solid evidence base in this area. Keyword and burst analyses reveal that the field has evolved from early mechanistic studies on oxidative stress and insulin resistance toward emerging themes, including clinical trials, cognitive impairment, and blood–brain barrier penetration. Notably, the increasing attention to blood–brain barrier-related topics highlights the growing recognition of central drug delivery as a key translational challenge. The emergence of mild cognitive impairment also signals an expanding research interest beyond motor symptoms, highlighting a key unmet need in non-motor symptom management.

### 4.2 GLP-1 receptor agonists and PD

Epidemiological studies have provided early clues for the potential neuroprotective effects of GLP-1 receptor agonists in the treatment of PD. For example, a national cohort study from Denmark showed that the risk of being diagnosed with PD within 10 years in patients who continuously used GLP-1 receptor agonists was approximately 43% lower than that in patients who used DPP-4 inhibitors (HR = 0.57) (Gamborg et al., 2025). Compared with patients using insulin, GLP-1 receptor agonist users also showed a trend toward lower disease onset risk. It is now widely recognized that type 2 diabetes mellitus significantly increases the risk of PD onset (Stockmann et al., 2025). This protective effect is based on the widespread expression of GLP-1R in the brain, which can be detected in regions such as the hypothalamus, tyrosine hydroxylase-positive (TH+) dopaminergic neurons in the substantia nigra pars compacta, and the hippocampus, with the densest distribution in the hypothalamus (Shimizu et al., 1987; Merchenthaler et al., 1999; Liu et al., 2024), providing a structural basis for the direct action of GLP-1 receptor agonists on degenerated targets. Through the limited penetration of the blood–brain barrier and peripheral-central signal transduction, GLP-1 receptor agonists can activate GLP-1R on the surface of neurons and exert multi-dimensional neuroprotective effects. In terms of neuronal nutritional support and anti-apoptosis, GLP-1 receptor agonists can upregulate the expression of BDNF in the brains of MPTP mice and enhance Akt activity, protecting motor function from neurotoxin damage (Ji et al., 2016). In terms of inhibiting neuroinflammation, GLP-1R activation can inhibit microglial over-activation, reduce the release of pro-inflammatory factors such as TNF-α and IL-6, and relieve the neuroinflammatory microenvironment. Similarly, exendin-4 has also been shown to significantly reduce microglial and astrocytic activation in the substantia nigra and striatum of MPTP-induced PD mice (Rimal et al., 2025). In terms of improving mitochondrial function and reducing oxidative stress, liraglutide can restore the mitochondrial fusion-fission dynamic balance induced by MPTP in mice, enhance autophagic flux, and reduce α-synuclein aggregation and oxidative stress levels (Lin et al., 2021). Similarly, semaglutide can restore tyrosine hydroxylase (TH) levels, reduce α-synuclein pathological deposition, and significantly alleviate chronic brain responses in the chronic MPTP model (Zhang et al., 2019). In addition, studies have confirmed that GLP-1R activation can affect aging-related cellular processes, manifested as enhanced mitochondrial function, increased cellular stress resistance, and reduced inflammation, and can regulate autophagy and reduce protein aggregation (Chavda et al., 2024; Hong et al., 2024). Therefore, the neuroprotective effect of GLP-1 receptor agonists may be partially attributed to their ability to delay aging-related neuronal damage. In summary, the above mechanisms together constitute the biological basis for GLP-1 receptor agonists to transition from metabolic regulation to neuroprotection. It is worth noting that in the α-synuclein preformed fibril (PFF)-injected preclinical mouse model of PD, an increase in α-synuclein pathological accumulation was observed in some sustained-release GLP-1 analogs (Bergkvist et al., 2021), suggesting that different drug structures and dosing regimens may have differential effects on PD pathology, further highlighting the necessity of individualized assessment.

### 4.3 Research trends

Current research on GLP-1 receptor agonists for PD treatment has gradually expanded from single-target mechanism verification to multi-target strategies, with “GIP/GLP-1 dual agonists” emerging as research hotspots and a growing trend. Although traditional single-target GLP-1 receptor agonists have shown neuroprotective effects in PD animal models, direct comparative studies within the same models have provided evidence supporting the superiority of multi-target approaches. Zhang et al. (2020) found that the dual-target GLP-1/GIP receptor agonist DA-CH5 was significantly superior to the single-target agonist liraglutide in preventing motor impairment, preserving substantia nigra dopaminergic neurons, and reducing neuroinflammation in the MPTP mouse model. In the rotenone-induced PD rat model, Delvadia et al. (2025) demonstrated that the dual-target agonist tirzepatide could significantly prevent motor deficits, upregulate striatal dopamine levels, and reduce α-synuclein aggregation, with a neuroprotective effect superior to that of the single-target exendin-4. Cell studies further confirmed that, in cell models induced by oxidative stress and apoptosis, the neuroprotective, antioxidant, and anti-inflammatory effects of the dual-target agonist tirzepatide and the triple-target agonist retatrutide were superior to those of the single-target exendin-4 (Kopp et al., 2024). In summary, through receptor cooperation and central permeability, multi-target strategies show superior neuroprotective effects to single-target drugs at both the animal and cellular levels, providing a more promising strategy for potential disease-modifying therapy in PD.

However, whether the neuroprotective effects of multi-target agonists can be fully translated into clinical efficacy depends crucially on their blood–brain barrier penetration efficiency and drug concentration in the target brain regions. Existing GLP-1 receptor agonists have large molecular weights, and their blood–brain barrier penetration is generally limited, primarily acting through indirect pathways such as peripheral–central signal transduction or circumventricular organs to exert their effects (Katsurada and Yada, 2016; Salameh et al., 2020). This structure-determined difference in central accessibility directly affects clinical outcomes. Christensen et al. (2015) found that the cerebrospinal fluid concentration of liraglutide was only 0.02% of the plasma concentration and showed no correlation with clinical efficacy. “Blood–brain barrier” has attracted sustained attention recently, indicating that improving central drug delivery has become a technological frontier in this field. The exendin-4-modified nano-iron chelator developed by Huang et al. (2024) namely Ex-4@DFO NPs, can cross the blood–brain barrier and enter the brain in the MPTP mouse model, significantly reducing dopaminergic neuron loss in the substantia nigra pars compacta and inhibiting microglial activation. The surface exendin-4 modification enables active targeted delivery to the lesion area by binding to GLP-1R, which is highly expressed under PD pathological conditions. Nanotechnology-based delivery approaches offer a key strategy for overcoming the blood–brain barrier penetration bottleneck of GLP-1 receptor agonists by enhancing central drug exposure and improving targeting specificity.

Meanwhile, the protection of cognitive function among non-motor symptoms is becoming an important research direction for GLP-1 receptor agonists in the treatment of PD. Keyword emergence detection shows that “mild cognitive impairment” is one of the themes with relatively high emergence intensity in recent years. PD-related cognitive decline is closely associated with brain insulin resistance and impaired hippocampal neurogenesis (Athauda et al., 2017; Hölscher, 2020), and GLP-1 receptor agonists may slow cognitive impairment by upregulating BDNF, improving hippocampal synaptic plasticity, and reducing neuroinflammation (During et al., 2003; Ji et al., 2016; Rimal et al., 2025). This mechanism has been preliminarily clinically verified in Alzheimer’s disease (AD) and mild cognitive impairment (MCI). The secondary analysis of the REWIND trial by Cukierman-Yaffe et al. (2020) showed that dulaglutide significantly reduced the risk of cognitive impairment (HR 0.86, 95% CI 0.79–0.95) in patients with T2DM after 5.4 years of follow-up, suggesting that GLP-1 signaling has a long-term protective effect on cognition. Further mechanistic studies have shown that GLP-1R activation can exert neuroprotective effects through two pathways. On the one hand, the cAMP/PKA/CREB signaling cascade can upregulate BDNF expression, enhance hippocampal synaptic plasticity, and promote memory consolidation (During et al., 2003); on the other hand, the PI3K/Akt pathway can inhibit GSK-3β overactivation, reduce abnormal tau phosphorylation and neurofibrillary tangle formation, thereby mitigating metabolic stress-induced neuronal damage (Dou et al., 2025). The above mechanisms provide a cross-disease theoretical basis for GLP-1 receptor agonists to improve PD-related cognitive impairment. However, evidence on the effects of GLP-1 receptor agonists on PD-related cognitive impairment remains limited. The study by Salem et al. (2025) found that semaglutide significantly reduced oxidative stress and inflammatory biomarkers in a rat model of diabetes combined with PD, and improved the cognitive, motor, and olfactory functions of the animals. Although the open-label trial of exenatide conducted by Aviles-Olmos et al. (2013) did not prespecify the primary cognitive endpoint, *post-hoc* analysis showed that the treatment group showed improvement over the control group on the Mattis Dementia Rating Scale, Second Edition (MDRS-2). However, neither of these studies was specifically designed for cognitive impairment, and the proof-of-concept trial of exenatide in MCI patients reported by Dei Cas et al. (2024) did not demonstrate a clear cognitive benefit, suggesting that disease stage, drug molecular structure, and degree of central exposure may be key variables influencing efficacy. Currently, there remains a scarcity of randomized controlled trials specifically targeting Parkinson’s disease-related cognitive impairment. Future studies should include cognitive function as a primary endpoint and explore the therapeutic potential of multi-target agonists with improved blood-brain barrier permeability in this population to validate the potential disease-modifying benefits of GLP-1 receptor agonists across various symptom domains.

### 4.4 Clinical evidence and study limitations

Existing clinical studies investigating GLP-1 receptor agonists in PD have reported inconsistent findings, and overall evidence remains heterogeneous (Athauda et al., 2017; Helal et al., 2025). Differences in pharmacological properties between short-acting and long-acting compounds (Drucker, 2018), variations in patient characteristics across trials (Meissner et al., 2024), and methodological heterogeneity in study design and outcome assessments may all contribute to these discrepancies. In particular, differences in disease stage, treatment background, and follow-up duration across studies limit direct comparability of results (Meissner et al., 2024). Therefore, current clinical evidence does not yet allow for definitive conclusions regarding the therapeutic effects of GLP-1 receptor agonists in PD (Helal et al., 2025). Further large-scale, well-designed randomized controlled trials are needed to clarify their potential clinical relevance.

### 4.5 Limitations

This study has several limitations. First, the analysis was restricted to the Web of Science Core Collection and Scopus databases; relevant publications indexed in other databases may have been missed. Second, bibliometric analyses depend on the completeness and consistency of citation data, which may introduce database-related and citation-based bias. Third, only English-language publications were included, introducing potential language bias. Fourth, although the search strategy was carefully designed and independently reviewed by experienced investigators, no formal PRESS assessment was performed. Fifth, the reliance on free-text terms and drug-specific keywords may have led to incomplete retrieval of studies using broader incretin-related terminology. Sixth, publication data for 2026 were incomplete at the time of analysis; therefore, trends for this year should be interpreted with caution and are not directly comparable with full-year data. Finally, as this field is rapidly evolving, bibliometric findings represent a time-specific snapshot of the literature and may not fully reflect the most recent developments. Taken together, these limitations should be considered when interpreting the results of this study.

## 5 Conclusion

Through bibliometric analysis, this study systematically mapped the research landscape and knowledge structure of GLP-1 receptor agonists in PD. Current research hotspots focus on oxidative stress, insulin resistance, and α-synuclein pathology, while emerging themes such as GIP/GLP-1 dual agonists, blood–brain barrier-related mechanisms, and mild cognitive impairment suggest expanding interest toward drug development, neuroprotection, and early intervention strategies. Overall, the field has evolved into a rapidly growing interdisciplinary area integrating metabolic and neurodegenerative research. However, the potential disease-modifying effects of GLP-1 receptor agonists in PD remain to be fully clarified, and further high-quality experimental and clinical studies are required. Future research should focus on multi-target therapeutic development, improved central nervous system delivery, and the identification of patient subgroups that may benefit most from these interventions, thereby advancing precision medicine approaches in PD.

## Data Availability

The original contributions presented in this study are included in the article/Supplementary material, further inquiries can be directed to the corresponding author.

## Footnotes

1
https://doi.org/10.17605/OSF.IO/U7WDY
