# Supplementary material for: Research hotspots and trends of GLP-1 receptor agonists in the treatment of Parkinson’s disease: a bibliometric analysis
Source: Front Neurosci. 2026 Jul 29;20:1894856. doi: 10.3389/fnins.2026.1894856 (PMC13462061; doi:10.3389/fnins.2026.1894856)
Supplement: Supplementary file 1 [file Data_Sheet_1.pdf]

Appendix 1: Search Strategy

Data sources	Search	Query	Results
Web of Science	#1	TS=("Parkinson's disease" OR "parkinson*")	251,003
	#2	TS=("Glucagon-Like Peptide-1 Receptor Agonists" OR "GLP-1 receptor agonist" OR "GLP-1 RA*" OR "GLP-1 analog*" OR "GLP-1 analogue*" OR "GLP-1R agonist" OR "incretin mimetic" OR exenatide OR liraglutide OR lixisenatide OR albiglutide OR dulaglutide OR semaglutide OR tirzepatide OR retatrutide OR nly01 OR Bydureon OR PT320 OR CagriSema OR Orforglipron OR Survodutide OR PB-119 OR DA5-CH OR DA-JC1 OR CT-996 OR Danuglipron OR Lotiglipron OR RGT-075 OR EXN1096)	26,232
	#3	1# AND 2#	344
Scopus	#1	TITLE-ABS-KEY ("Parkinson's disease" OR "parkinson*")	37,936
	#2	TITLE-ABS-KEY ("Glucagon-Like Peptide-1 Receptor Agonists" OR "GLP-1 receptor agonist" OR "GLP-1 RA*" OR "GLP-1 analog*" OR "GLP-1 analogue*" OR "GLP-1R agonist" OR "incretin mimetic" OR exenatide OR liraglutide OR lixisenatide OR albiglutide OR dulaglutide OR semaglutide OR tirzepatide OR retatrutide OR nly01 OR Bydureon OR PT320 OR CagriSema OR Orforglipron OR Survodutide OR PB-119 OR DA5-CH OR DA-JC1 OR CT-996 OR Danuglipron OR Lotiglipron OR RGT-075 OR EXN1096)	37,936
	#3	1# AND 2#	638
